# High Level Bioaerosol Protection against Infective Aerosols: How Medical Face Masks Compare against Respirators

**DOI:** 10.1155/2022/6978661

**Published:** 2022-10-22

**Authors:** Christian M. Sterr, Aline Dick, Lena Schellenberger, Julian Zirbes, Claudia Nonnenmacher-Winter, Frank Günther

**Affiliations:** Division of Infection Control and Hospital Epidemiology, Philipps University Marburg, Marburg, Germany

## Abstract

Face masks and respirators are commonly used to prevent the transmission of infectious diseases that spread by respiratory droplets and aerosols. However, there is still uncertainty about the protective effect of the different types of masks against virus containing aerosols. To determine the as-worn bioaerosol protection efficacy of different face coverings and estimate the possible protective function against airborne diseases, we challenged different respirators and medical masks on a standardized dummy head with a bioaerosol containing MS2 bacteriophages as virus surrogates. In our experiments, FFP2 respirators showed the highest filtration efficacy 94 ± 4 (SD) % followed by medical masks 93 ± 3 (SD) % and KN95 respirators 90 ± 7 (SD) %. Nevertheless, we found no statistically significant difference between respirators and medical masks in terms of provided protection against infective aerosols. Our findings indicate that both respirators and medical masks provide a high as-worn bioaerosol protection efficacy against virus containing aerosols, and therefore, a very high protection against airborne diseases. Considering the higher comfort, better availability, and lower price of medical masks in contrast to respirators, it is recommendable to use medical face masks especially in low risk situations and in general public.

## 1. Introduction

Face masks and respirators have been used to prevent the transmission of infectious diseases that spread by respiratory droplets and aerosols in hospital settings for years [1]. With the exception of East Asia, mask usage by the general public was not common globally until the onset of the SARS-CoV-2 pandemic in early 2020 [2].

During the pandemic, people started using different types of masks including respirators, surgical masks, and cloth masks every day to conform to the masking policy of their respective regions.

While recent randomized controlled trials (RCTs) could not find an advantage of respirators over medical masks in terms of protection against respiratory infections, in some countries, respirators even became compulsory in public transport and shops [3–5].

Generally, wearing masks seems to stop the virus from spreading. In a hair stylist salon in the US with a mandatory face covering policy for clients and employees, after having been serviced by two symptomatic and COVID-19 positive hair dressers, there was no reported symptomatic COVID-19 disease among 139 clients within 14 days of follow-up [6].

In Arizona, USA, a state-wide analysis of around 1,000 public schools on the odds of an outbreak at school depending on the respective masking policy showed that an outbreak was 3–5 times less likely in schools with mandatory masking compared to those with no mandatory masking policy [7].

Originally, the various types of face coverings were intended for different situations.

Respirators (e. g. FFP2, FFP3) were designed to protect workers from dust or technical aerosols in an occupational environment [8]. Nowadays, occupational health and safety regulations recommend the usage of these devices for health care workers (HCW) to provide highest possible protection against airborne diseases. In contrast, respirators are not tested for the protective function against biological agents in standard [1, 8].

Medical or surgical face masks are designed to protect patients from HCWs [9]. These masks are tested in a different setting only for the filtration efficacy against a *Staphylococcus aureus* containing bio aerosol [9]. The respective standard only demands a material test without considering a possible fit factor [9]. Cloth masks and other face masks are usually not tested according to existing standards. Therefore, their protective function is only presumptive and varies widely depending on the materials used [10].

Although the protective function of respirators is believed to be higher than that of medical face masks, different types of face coverings are usually tested under different circumstances, and thus, cannot be compared directly [8, 9]. Studies that actually did compare respirators and surgical face masks could only find little or no differences under clinical as well as under *in vitro* conditions. To evaluate the protection against influenza provided by N95 respirators and surgical masks in a hospital setting, Loeb et al. randomly assigned masks and respirators to two groups of around 200 HCW each. They found that the protective effect of surgical masks and N95 respirators against influenza infection were similar [11].

An *in vitro* study conducted previously in our lab showed that the as-worn protective effect of surgical masks and respirators against a test aerosol on a dummy head was similar [10].

Despite these findings, the extent of protective effect of the different types of face coverings under comparable conditions is unclear. There are no established standards or previous population-based study reports in this regard. As a result of that, many decision makers in health care still claim that there was not enough evidence.

By using a viral aerosol, we took into account the infectivity of the virus as an additionally relevant parameter. Passage through the masks might impede the virus integrity and potentially dries it out. Therefore, virus particles may penetrate through the mask, but consequently lose their ability to infect host cells. It was the aim of our study to substantiate the clinical data mentioned above with in vitro data from tests with bioaerosols.

To determine the as-worn bioaerosol protection of different face coverings against an infective aerosol under similar circumstances and to estimate the possible protection function against airborne diseases, we challenged different respirators and medical face masks on a standardized dummy head with an MS2 bacteriophage-containing virus laden bioaerosol.

## 2. Methods

The number of infective bacteriophages was measured before fumigation and behind the dummy head, using a double-layer plaque assay as described below. As a control, experiments in the test rig were conducted without any masks mounted on the dummy head.

### 2.1. Masks

Masks intended for our experiments were assigned to three groups. Group 1: FFP2 respirators tested according to DIN 149; group 2: KN95 respirators; and group 3: surgical masks tested according to DIN 14683.

### 2.2. Culture Media and Solutions

In our experiments, we used a double-layer plaque assay for the detection of the bacteriophages. As a bottom layer, we used tryptone-yeast extract-glucose agar (TYGA) (Trypticase peptone 10 g, yeast extract 1 g, NaCl 8 g, agar 20 g, distilled water 1000 mL). After dilution in boiling water, the agar-solution was sterilized at 121°C for 15 minutes and the pH-value was adjusted to 7.2 at 25°C. As a top layer, we used DEV nutrient agar (Merck, Darmstadt, Germany). For the dilution of the phages, we used a calcium-glucose solution (3 g CaCl2^*∗*^2H20, 10 g glucose, 100 mL distilled water) that was filtered through a 0.22 *μ*m sterile filter after preparation. We further used a peptone saline solution for diluting the phages (Peptone 1.0 g, sodium chloride 8.5 g, distilled water 1000 mL). PH-value of the Peptone saline solution was adjusted to 7.0 at 25°C after sterilization at 121°C for 15 minutes.

### 2.3. Aerosol

**Figure 1:**
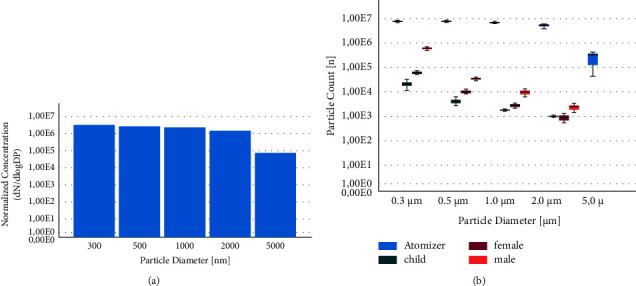
(a) Size distribution of the applicated bioaerosol. (b) Particle count of used aerosol compared to human exhalation samples.

*Escherichia* phages MS2 ATCC 15597-B1 were obtained from the Deutsche Sammlung für Mikroorganismen und Zellkulturen (Braunschweig, Germany) and propagated in *Escherichia coli* cultures (ATCC 5210) before stored as 1 mL aliquots containing 1^*∗*^10^9^ plaque forming units (pfu) at −80°C. For the experiments, a 1 mL aliquot containing the phages was thawed and diluted in 9 mL of peptone saline solution before being atomized using the AGK 2000 atomizer (Palas GmbH, Karlsruhe, Germany). The produced aerosol showed a particle size distribution from 0.3 to 5 *μ*m (Figure 1(a)) measured by a particle scanner using a light scattering technique, Abakus Air (Markus Klotz, Bad Liebenzell, Germany). Channels measured were 0.3 *μ*m, 0.5 *μ*m, 1.0 *μ*m, 2.0 *μ*m, and 5.0 *μ*m.

### 2.4. Test Rig

**Figure 2:**
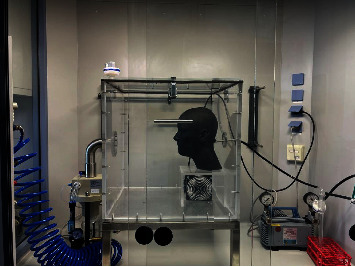
A practical mask test rig with an atomizer (left), a particle-tight chamber with a standardized test head (middle), an impinger and a vacuum pump (right).

To assess the as-worn bioaerosol protection efficacy against a virus laden aerosol of the different face masks in a realistic way, we used our established test rig as described previously [10]. In brief, masks were mounted on a standard sized dummy head [12], which was placed into an acrylic glass chamber. As no sealant was used, some leakage may have occurred since some masks did not fit airtight. In contrast to our previous test system, the described virus laden-aerosol was conducted into the chamber instead of a noninfectious test aerosol substance. Hence, the experiment was conducted under a laminar airflow bench. To achieve a homogeneous distribution of the aerosol, a small ventilator was placed into the chamber. The artificial trachea was connected to an impinger containing 10 mL of a peptone saline solution to collect the “inhaled” virus containing aerosol. To create a steady airflow of 10–12 l/min, we connected a vacuum pump behind the impinger with a flowmeter as intermediary (Figure 2).

### 2.5. Detection of Bacteriophages

After finishing an experiment at the test rig, a dilution series was performed using 120 *μ*L of the virus containing solution from the impinger and 1080 *μ*L peptone saline stock solution. Of the dilution factors 10^−6^ and 10^−7^, 1080 *μ*L were mixed with 25 *μ*L of a calcium-glucose, 100 *μ*L of an *E. coli*-containing brain heart infusion solution, and 3 mL DEV-Agar. After mixing, this top layer composition was poured onto the bottom layer TYGA-plates and incubated for 18 +/− 2 h at 36°. Plaques were counted and considered for calculation, if there were between 30 and 300 pfu per plate. To determine the reduction factor of a mask, a second dilution series with subsequent plaque counting was performed as described with the solution from the atomizer. In contrast to the first series, in this second series dilution factors 10^−4^ and 10^−5^ were used for the top layer composition as preliminary experiments showed a two log scale loss of infectivity due to the test rig. Measurements of the test rig without a mask on the dummy head were performed as a control to calculate the as-worn bioaerosol protection of the masks.

(1) $$\begin{array}{c} Bioarerosol\;Protection=\frac{\sum Mean\;mask\left(\left(plaque\;count\;impinger\right)/\left(plaque\;count\;atomizer\right)\right)}{\sum Mean\;control\left(\left(plaque\;count\;impinger\right)/\left(plaque\;count\;atomizer\right)\right)}\text{.} \end{array}$$

### 2.6. Human Exhalation Measurements

To contextualize the measured as-worn bioaerosol protection, we exemplarily collected human expiratory samples from men and women volunteers, who were between 3 and 50 years of age. Test persons were equipped with an oxygen mask and asked to breathe out for 20 seconds by constantly intonating the vowel a to ensure that the aerosol-laden airflow can flow out through the mouth largely unimpeded while the particle concentration in the respiratory flow were counted continuously with a particle counter, Abakus Air (Markus Klotz, Bad Liebenzell, Germany).

### 2.7. Statistical Methods

Statistical analysis and graphing was performed using SPSS 28. (IBM, Armonk, USA) by Kruskal-Wallis test and ANOVA at a significance level of 0.05.

## 3. Results

**Figure 3:**
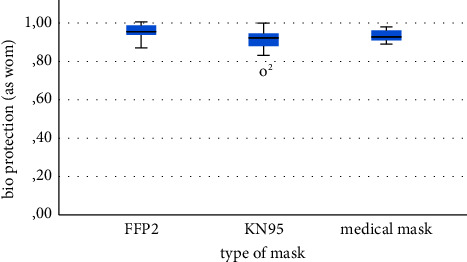
Distribution of as-worn bioaerosol protection against infective MS2-containing aerosol.

FFP2 respirators showed the highest reduction by reducing the number of infective plaques to about 94 ± 4 (SD) % (*n* = 6) compared to the controls. Medical masks 93 ± 3 (SD) % (*n* = 11) and KN95 respirators 90 ± 7 (SD) % (*n* = 9) still showed a very high reduction (Figure 3). Nevertheless, these differences were not statistically significant.

By comparing the test aerosol with human exhalation samples we were able to demonstrate, that the particle concentration used in our experiments was much higher than the concentration of particles of all sizes exhaled by humans (Figure 1(b)). Furthermore, we found that men release about four times more aerosol particles than women. In children between the ages of 3 and 6 years, the aerosol release in the respiratory stream is detectable just slightly above the aerosol load in the ambient air.

## 4. Discussion

Our findings revealed that both respirators and medical masks provide a high as-worn bio protection function against virus containing aerosols, and therefore, a very high protection against transmission of airborne diseases. In the experiments, respirators as well as medical masks reduced the number of infective MS2 bacteriophages by more than 90%.

These findings stand in line with recent data from meta-analysis of different RCTs that compared the effectiveness of N95 respirators and surgical masks against infections with SARS-CoV-2 in low risk situations [3–5]. Bartoszko et al. analyzed four RCTs and reported that wearing medical masks was not associated with a higher risk of respiratory illness or infection compared to N95 respirators [4]. Barycka et al. also reported that respirators and medical masks provided comparable protection [3].

However, occupational health and safety regulations still recommend the usage of respirators for HCW and many countries, such as Germany, even demand to wear respirators in public low-risk situations such as visiting shopping centers to conform with their mandatory masking policy [1].

Our study enables the direct comparison of respirators and medical mask under similar conditions. Therefore, our experiments underpin the findings reported above with results from a different perspective *in vitro*. Although, it is limited because of a small sample size.

In our former experiments, we demonstrated a considerably lower filtration efficacy for the tested respirators and medical masks of about 41–65% as-worn filtration efficacy compared to over 90% bioaerosol protection in our recent findings [10]. Although we had used the same test rig, this difference seems reasonable for a couple of reasons.

First, in our former study, the measurements were taken at an aerosol diameter of 0.5 *μ*m [10]. At that particle size, leakage through the filtration material of the respective face coverings is known to be very high [13]. The viral aerosol used in this study had a different particle size distribution (Figure 1(a)) compared to our former study. Particles of larger size are usually better filtrated by the same material [14]. That might be one explanation for differences in the measured filtration function.

However, although SARS-CoV-2 is only about 60–140 nm in diameter and MS2 bacteriophages are slightly smaller, experiments with a broader size distribution and larger particles are closer to realistic conditions in daily life [15–17]. This is because small virus particles are usually agglutinated to form larger particles consisting of water, cells, and proteins [15, 17].

Our human exhalation measurements confirmed the presence of a size distribution from 0.3 to 2.0 *μ*m among exhaled particles. We could further demonstrate that the particle concentration used in our experiments was much higher than the number of exhaled human particles for all particle sizes (Figure 1(b)). Additionally, exhaled human particles are greatly diluted in real life low risk situations. Considering a minimum infectious dose of about 100 particles for SARS-CoV-2, the archived bio protective effect in everyday situations will be higher, than the reported as-worn bioaerosol protection in our study.

By using a viral aerosol, we further took into account the infectivity of the virus as an additionally relevant parameter. Although MS2 bacteriophages are more resistant to environmental conditions than SARS-CoV-2 because of their missing capsule, the maintenance of infectivity is influenced by similar conditions and factors [18]. Passage through the masks impedes the virus integrity and potentially dries it out. Therefore, virus particles may penetrate through the mask, but consequently lose their ability to infect host cells. This realistic situation can be captured by our actual experimental setup, while a rather worst-case scenario with mask passage of aerosols of noninfectious particles or liquids was the focus of our former study [10].

Aside from the filtration efficacy, there are other important factors to be considered when comparing medical masks and respirators. Face coverings only work well, if worn properly. It is out of the question that respirators induce significantly more discomfort than medical masks [19]. A reason for that is the two-to three-fold higher airflow resistance [10, 19]. Hence, this leads to a lower user adherence and consequently a lower overall protection rate.

In conclusion, both respirators and medical masks provide a high filtration efficacy against virus containing aerosols in situations with low physical exertion and therefore a very high protection against airborne diseases. Considering the higher comfort, better availability, and lower price of medical masks compared to respirators, it is recommendable to use medical masks in low risk situations and in general public.

## Data Availability

The data that support the findings of this study are available from the corresponding author upon reasonable request.
